# The Rattlesnake

**Published:** 1889-09

**Authors:** 


### THE RATTLESNAKE.

From a profusely illustrated article by Dr. S. Wier Mitchell, on "The Poison of Serpents," in the August *Century*, we quote the following: "Let us observe what happens when the rattlesnake means mischief. He throws himself into a spiral, and about one-third of his length, carrying the head, rises from the coil and stands upright. The attitude is fine and warlike, and artists who attempt to portray it always fail. He does not pursue, he waits. Little animals he scorns unless he is hungry, so that the mouse or the toad he leaves for days unnoticed in his cage. Larger or noisy creatures alarm him. Then his head and neck are thrown far back, his mouth is opened very wide, the the fang held firmly erect, and with an abrupt swiftness, for which his ordinary motions prepare one but little, he strikes once and is back on gaurd again, vigilant and brave. The blow is a stab, and is given by throwing the head foward while the half-coils below it are straightened out to lengthen the neck and give power to the motions which drive the fangs into the opponent's flesh; as they enter, the temporal muscle closes the lower jaw on the part struck, and thus forces the sharp fang deeper in. It is a thrust aided by a bite. At this moment the poison duct is opened by the relaxation of the muscle which surrounds it, and the same muscle which shuts the jaw squeezes the gland, and drives its venom through the duct and hollow fang into the bitten part.

"In so complicated a series of acts there is often failure. The tooth strikes on tough skin and doubles back or fails to enter, or the serpent misjudges distance and falls short and may squirt the venom four or five feet in the air, doing no harm. I had a curious experience of this kind in which a snake eight fee six inches long threw a teaspoonful or more of poison athwart my forehead. It missed my eyes by an inch or two. I have had many near escapes, but this was the grimmest of all. An inch lower would have cost me my sight and probably my life.

"A snake will turn and strike from any posture, but the coil is the attitude always assumed when possible. The coil acts as an anchor and enables the animal to shake its fangs loose from the wound. A snake can rarely strike beyond half his length. If both fangs enter, the hurt is doubly dangerous, because the dose of venom is doubled. At times a fang is left in the flesh, but this does not trouble the serpent's powers as a poisoner, since numberless teeth lie ready to become firmly fixed in its place, and both fangs are never lost together. The nervous mechanism which controls the act of striking seems to be in the spinal cord, for if we cut off a snake's head and then pinch its tail, the stump of the neck returns and with some accuracy hits the hand of the experimenter—if he has the nerve to hold on. Few men have. I have not. A little Irishman who took care of my laboratory astonished me by coolly sus taining this test. He did it by closing his eyes and so shutting out for a moment the too suggestive view of the returning stump. Snakes have always seemed to me averse to striking, and they have been on the whole much maligned.

"Anv cool, quiet person moving slowly and steadily may pick up and handle gently most venomous serpents. I fancy, however, that the vipers and the copperheads are uncertain pets. Mr. Thompson, the snake keeper at the Philadelphia Zoölogical, handles his serpents with impunity; but one day having dropped some little moccasins a few days old down his sleeve while he carried their mamma in his hand, one of the babies bit him and made an ugly wound. At present the snake staff is used to handle snakes.

"I saw one October, in Tangiers, what I had long desired to observe—a snake charmer. Most of his snakes were harmless; but he refused, with well-acted horror, to permit me to take hold of them. He had also two large brown vipers; these he handled with care, but I saw at once that they were kept exhausted of their venom by having been daily teased into biting on a bundle of rags tied to a stick. They were too tired to be dangerous. I have often seen snakes in this state. After three or four fruitless acts of instinctive use of their venom they give up, and seem to become indifferent to approaches, and even to rough handling."

